# Regulation of the Cyanobacterial CO_2_-Concentrating Mechanism Involves Internal Sensing of NADP^+^ and α-Ketogutarate Levels by Transcription Factor CcmR

**DOI:** 10.1371/journal.pone.0041286

**Published:** 2012-07-20

**Authors:** Shawn M. E. Daley, Anthony D. Kappell, Marla J. Carrick, Robert L. Burnap

**Affiliations:** 1 Microbiology and Molecular Genetics, Oklahoma State University, Stillwater, Oklahoma, United States of America; 2 Department of Biochemistry & Molecular Biology, Oklahoma State University, Stillwater, Oklahoma, United States of America; Max Planck Institute for Chemical Ecology, Germany

## Abstract

Inorganic carbon is the major macronutrient required by organisms utilizing oxygenic photosynthesis for autotrophic growth. Aquatic photoautotrophic organisms are dependent upon a CO_2_ concentrating mechanism (CCM) to overcome the poor CO_2_-affinity of the major carbon-fixing enzyme, ribulose-bisphosphate carboxylase/oxygenase (Rubisco). The CCM involves the active transport of inorganic forms of carbon (C_i_) into the cell to increase the CO_2_ concentration around the active site of Rubisco. It employs both bicarbonate transporters and redox-powered CO_2_-hydration enzymes coupled to membranous NDH-type electron transport complexes that collectively produce C_i_ concentrations up to a 1000-fold greater in the cytoplasm compared to the external environment. The CCM is regulated: a high affinity CCM comprised of multiple components is induced under limiting external Ci concentrations. The LysR-type transcriptional regulator CcmR has been shown to repress its own expression along with structural genes encoding high affinity C_i_ transporters distributed throughout the genome of *Synechocystis* sp. PCC 6803. While much has been learned about the structural genes of the CCM and the identity of the transcriptional regulators controlling their expression, little is known about the physiological signals that elicit the induction of the high affinity CCM. Here CcmR is studied to identify metabolites that modulate its transcriptional repressor activity. Using surface plasmon resonance (SPR) α­ketoglutarate (α-KG) and the oxidized form of nicotinamide adenine dinucleotide phosphate (NADP^+^) have been identified as the co-repressors of CcmR. Additionally, ribulose­1,5­bisphosphate (RuBP) and 2­phosphoglycolate (2­PG) have been confirmed as co-activators of CmpR which controls the expression of the ABC-type bicarbonate transporter.

## Introduction

Mechanisms to concentrate inorganic carbon (C_i_) in the vicinity of the major carbon-fixing enzyme, ribulose-bisphosphate carboxylase/oxygenase (Rubisco), are often crucial for sustaining high rates of oxygenic photosynthesis. Cyanobacteria have evolved the capacity to overcome low ambient C_i_ concentrations by actively acquiring C_i_ in the form of bicarbonate (HCO_3_^−^) or by converting dissolved carbon dioxide (CO_2_) to HCO_3_^−^. Either way, C_i_ mainly in the form of HCO_3_^−^, is accumulated in the cyanobacterial cytoplasm. The operation of the C_i_ uptake systems allows the increase of the cytosolic levels of C_i_ to 1000-fold greater than extracellular levels [1]–[5]. This enables the high flux conversion of the inorganic carbon into organic carbon via the Calvin-Basham-Benson (CBB) cycle. The carbon-fixing enzyme of the CBB, Rubisco, is sequestered within a specialized protein microcompartment termed the carboxysome that is located in the cytoplasm in cyanobacteria. The carboxysome is bounded by a protein shell considered to be selectively permeable to key metabolites including HCO_3_^−^. Besides Rubisco, the carboxysome also contains carbonic anhydrase. Consequently, any HCO_3_^−^ diffusing into the carboxysome is efficiently dehydrated thereby increasing the local concentration of CO_2_, the actual substrate of Rubisco. These adaptations function to overcome the notoriously poor selectivity of Rubisco for CO_2_ over the more abundant, but non-productive competitive substrate, O_2_. Under low CO_2_ conditions, the oxygenase activity of Rubisco thus tends to increase, resulting in oxygenation, rather than carboxylation, of the substrate RuBP. This leads to the metabolically wasteful production of the two-carbon compound, 2-phosphoglycollate (2-PG), which needs to be salvaged in the process termed photorespiration. The accumulation of bicarbonate in the cytoplasm and operation of the carboxysome are absolutely required to avoid these wasteful processes and are collectively called the CO_2_-concentrating mechanism (CCM).

Several different C_i_ uptake systems have been identified in cyanobacteria, each with distinctive uptake flux capacity, and net affinity characteristics. Although the systems are mechanistically diverse, they nevertheless fall into two broad kinetic categories: lower affinity/high flux and higher affinity/low flux systems. While grown under high inorganic carbon (HC) conditions, where C_i_ is sufficient, cells typically express only the low-affinity/high flux transport activity, whereas the higher affinity/low flux systems are additionally expressed upon imposition of low inorganic carbon (LC) conditions. In *Synechocystis*, the basal level of C_i_ transport activity is related to the expression of the constitutive lower affinity/high flux C_i_ transporters: a Na^+^­dependent HCO_3_^­^ transporter BicA encoded by ORF *sll0834* [6] and the redox-driven CO_2_ uptake system NDH­I_4_ based on a specialized NDH-I complex encoded by the genes *ndhF4* (*sll0026*), *ndhD4* (*sll0027*), and *cupB* (*slr1302*) [7], [8]. Note that, *Synechocystis* ORF designations given in parentheses (e.g. slr1594). These complexes are intriguingly proposed to operate as ‘vectorial carbonic anhydrases’ catalyzing the hydration of CO_2_ and driven by the formation of a alkaline microdomain in the region of the CO_2_ hydration reaction [1]. The inducible C_i_ transporters that show increased expression upon shift from HC conditions to LC conditions are the high affinity HCO_3_^−^ transporter, BCT1, encoded by the *cmpAB(porB)CD* operon (*slr0040-44*; hereafter the *cmp* operon) [9], the high affinity Na^+^-dependent HCO_3_^−^ transporter, SbtA/B, encoded by *slr1512* and *slr1513* [10], [11], and redox-driven high affinity CO_2_ uptake system NDH-I_3_ encoded by the genes *ndhF3* (*sll1732*), *ndhD3* (*sll1733*), *cupA* (*sll1734*), and *sll1735* [7], [8], [12]–[14]. At the proteomic level, the induction is very striking, with the induced transporters accumulating as major fractions of the cellular complement of membrane proteins [14]. Functionally, this corresponds to increased CCM activity, increased affinity of Ci transport, and high overall photosynthetic efficiency even under relatively low ambient Ci conditions.

While considerable progress has been made in defining the structural genes required for the CCM, less information is available regarding their regulation. Importantly, the metabolic signals for the induction remain obscure despite considerable efforts to reveal them. The transcriptional regulators, CmpR (Sll0030), CcmR (aka NdhR, Slr1594), and Sll0822 are implicated in the control of expression of the low carbon (LC) inducible genes of the CCM [11], [15]–[18]. CmpR and CcmR exhibit homology to CbbR, a LysR family transcriptional regulator of the CO_2_ fixation genes in chemoautotrophic and anoxygenic photoautotrophs [19]. Sll0822 is a member of the AbrB family of transcriptional regulators and appears to function as a repressor of the expression of NDH-I_3_ and SbtA [18]. CmpR functions as a transcriptional activator and has been shown to increase the expression of the C_i_ responsive *cmp* operon encoding the BCT1 transporter during C_i_-limiting conditions in *Synechocystis* and *Synechococcus* PCC 7942 [16]. CmpR from *Synechococcus* PCC 7942 has been shown to bind a regulatory region upstream of the *cmp* operon using electrophoretic mobility shift assays and that the presence of the small molecules ribulose-1,5-bisphosphate (RuBP) or 2­phosphoglycolate (2-PG) enhanced binding [20]. The finding that 2-PG is involved in regulation of the induction of the CCM validates earlier suggestions that this might be the case [21] and is consonant with recent metabolomic analyses [22], [23].

**Figure 1:**
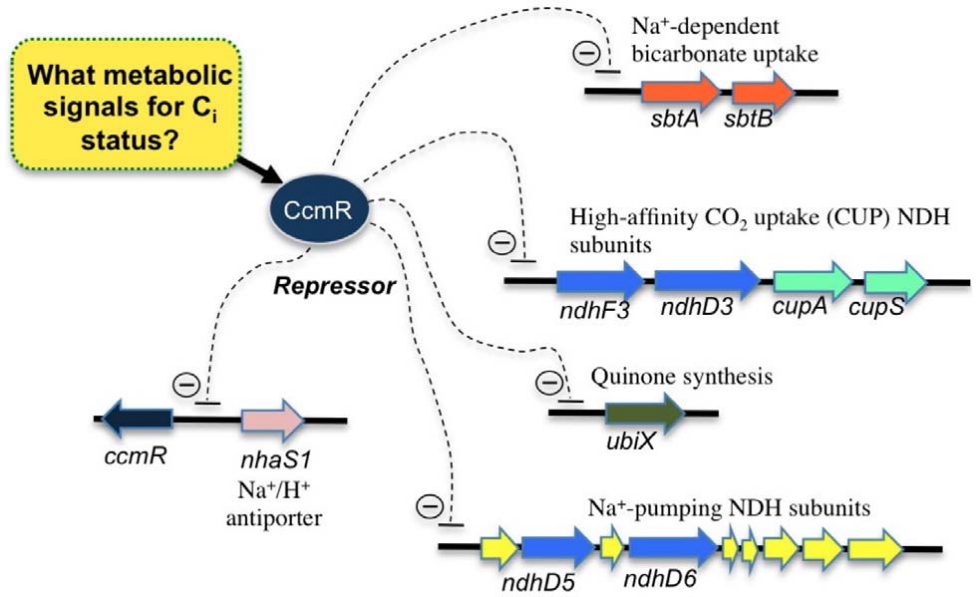
Organization of genes of the inducible high affinity CCM that are repressed by the LysR-type transcriptional regulator, CcmR of *Synechocystis* sp. PCC6803 [**11**]. Identification of the CcmR binding sites on DNA regulatory sequences constituting the operator regions has been performed for the *ccmR* and *ndhF3* genes [17]. This formed the basis for the investigation of the metabolic signals modulating CcmR repression performed in this study.

In *Synechocystis*, the other CbbR homolog, CcmR, acts as a negative regulator of CO_2_ responsive genes including the C_i_ transporters, NDH-I_3_ and SbtA [11], [17]. Although the deletion of the gene encoding CcmR is sufficient to cause the de-repression of genes for the high affinity C_i_ transporters in *Synechocystis* sp. PCC6803, the regulation appears to be complex an protein in the AbrB family of transcriptional regulators also appears to function as a repressor of the expression of NDH-I_3_ and SbtA [18]. CcmR in *Synechococcus* sp. PCC 7002 acts as a negative regulator for all the known CO_2_ responsive genes including the *ndh-I_3_* (aka, cup *chp*), *sbt*, and *bic* genes in that organism [15]. CcmR appears to be absent from the genome of *Synechococcus* PCC 7942, suggesting that CmpR or a yet unidentified regulator is responsible acts as a regulator of its complement of the genes encoding the SbtA and NDH-I_3_ transporters [5]. Microarray and mutational analysis of *Synechocystis* identified members of the CcmR regulon (Figure 1) consist of the gene clusters *sbtA*/*sbtB* (hereafter *sbt* operon), *ndhF3*/*ndhD3*/*cupA*/*sll1735* (hereafter *ndh-I_3_* operon), *slr2006*/*ndhD5*/*ndhD6*/*slr2009*/*slr2010*/*ssr3409*/*ssr3410*/*slr201*/*slr2012/slr2013* (hereafter *mnh* operon) and the genes *ccmR* and *ubiX* [11].

Here we describe the physical interaction between the repressor, CcmR and the DNA control regions of the *ndh-I_3_* operon and *ccmR*, which are chromosomal locations that were previously demonstrated to bind CcmR [17]. It is shown that the molecular mechanism controlling the CcmR with its DNA targets involves the binding of metabolic intermediates NADP^+^ and α-ketoglutarate (α-KG), which enhance binding of CcmR to repressor-binding sequences and thereby appear to act as co-repressors. This is first information on the metabolic signal responsible for the induction of the major CCM genes in *Synechocystis* and provides a mechanism for the de-repression of CCM genes in response to C_i_ limitation and the coordination of this process with the observed concomitant down-regulation of nitrogen acquisition genes. Additionally, we confirm that RUBP and 2-OG act as the ligand molecules for the other CbbR homolog, CmpR (Sll0030) from *Synechocystis* which is consistent with previous findings on the effectors of CmpR from *Synechococcus* PCC 7942 [20]. Taken together, the findings enable the formulation of a specific model for the metabolic control for adaptation to CO_2_-limiting conditions that is consistent with many previous physiological and molecular genetic experiments.

## Results

### Surface Plasmon Resonance illustrates the binding characteristics of CcmR to DNA fragments bearing the upstream region of members of the CcmR regulon

**Figure 2:**
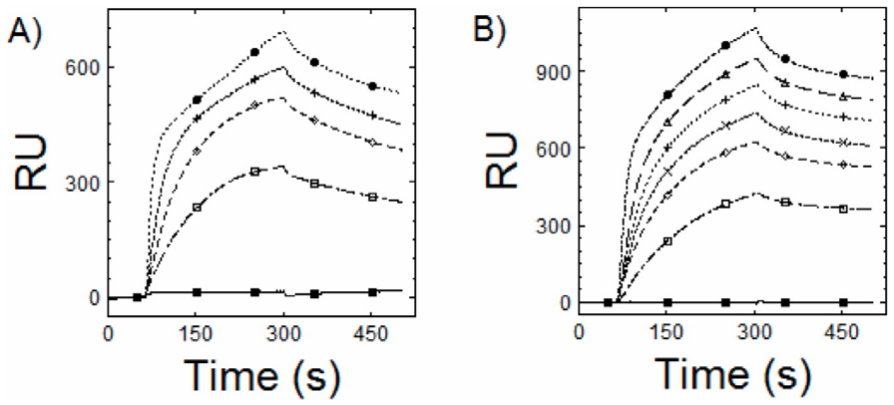
Surface Plasmon Resonance curves illustrating binding of CcmR to promoter regions of the *ccmR* and *ndhF3*. Biotinylated upstream duplex DNA ∼150 bp of the pccmR (A) and (B) pndhF3. Each DNA fragment was immobilized to a Neutravidin-coated SPR chip (Nomadics). The upstream sequences for *ccmR* and *ndhF3* that bind CcmR has been previously determined [17], (see Data S1 for primers used to generate DNA fragments). Heterologously expressed CcmR concentration for each target is as follows, markers are for visualization only; 0 nM (Closed Square), 250 nM (Open Square), 500 nM (Open Diamond), 750 nM (X), 1000 nM (+), 2000 nM (Open Triangle), 3000 nM (Closed Circle).

Previous work had mapped promoter DNA sequences that interacted with CcmR for two members of the CcmR regulon, *ndhF3* (first gene of the *ndh-I_3_* operon) and *ccmR* [17]. CcmR is a LysR-type transcriptional regulator (LTTR), which regulatory proteins that are generally observed to induce DNA bending in promoter regions and change their DNA binding characteristics depending upon the binding of small effector molecules that serve to modulate the activity of the LTTR in response to changes in metabolism [reviewed in [24]]. To characterize the binding of CcmR to defined chromosomal targets, surface plasmon resonance (SPR) was employed. SPR is an optical method of detecting interactions between an injected free biomolecule flowing over an immobilized biomolecule on the surface of a biosensor. The technique is based on the fact that when light strikes the surface of a thin layer of gold at a certain angle it is able to excite plasmons on the opposite side of the metal surface thereby generating an evanescence field [25]. The loss of reflected photons at a specific set of angles from the light striking the surface of the metal is reported as response units (RU) and is dependent principally on the mass of biomolecule bound to the surface, but also on the refractive index of the biomolecule immobilized on the metal surface and the interaction with the injected free biomolecule along the flow path within the evanescence field. Using SPR, the double stranded DNA fragments of *ccmR* and *ndhF3* that bind CcmR were tested to determine binding characteristics of heterologously expressed CcmR (Figure 2). The surfaces of separate SPR biosensors were prepared by immobilizing biotinylated-duplex DNA fragments containing each one the different upstream regions of the putative CcmR regulon. The immobilization involved a commercially prepared Neutravidin coating the surface of the SPR biosensor allowing high affinity binding of the biotinylated DNA to the biosensor surface. The upstream sequences for *ccmR* and *ndhF3* that bind CcmR that had been previously determined [17] and are within the corresponding immobilized DNAs on their respective sensors. Figure 2 shows the binding curves that result from the passage of CcmR protein over the immobilized promoter region DNA. In this set of experiments, CcmR has been introduced, at the 60 second time point, into the buffer flowing over the surface of the sensor and the CcmR-containing buffer flow continues until the 360 second time point. During this injection phase there is an accumulation of mass on the surface of the sensor chip reflected as the increase in RUs. After 360 seconds buffer flow is switched to buffer lacking CcmR so that what is observed is the gradual loss of mass from the biosensor surface. Increasing concentrations of CcmR (0 to 3000 nM) were injected into the flow path of the biosensor with immobilized DNA fragments of the upstream region of *ccmR* and *ndhF3* that bind CcmR causing an increase in RU (Fig. 2). The response curve during the association phase (60 to 360 sec) and dissociation phases (361 to 500 sec.) showed multiphasic increases and decreases in RU, respectively, at lower concentration of CcmR. At higher concentrations of CcmR, the response curves during the association phase were without reaching saturation of signal and the dissociation phase shows an initial drop in signal followed by a slow decrease in RU. Such complexity likely reflects multimeric binding and DNA bending changes that accompany LTTR-DNA interactions [26]–[29]. By comparison, the interaction of CcmR with non-specific duplex DNA (not shown), exhibited an ostensibly more rapid hyperbolic association phase indicating a more simple DNA protein interaction even though the specific DNA interaction of CcmR with cognate promoter region out-competes a 10-fold excess of non-specific DNA (see supplemental data file, Data S1, Figure S2). Because the complexity of the CcmR-promoter interaction, it was not possible to obtain good kinetic fits using a standard Langmuir isotherm model [30], [31] to determine the kinetic constants for the association (k_a_) and dissociation phase (k_d_). Nevertheless, specific effector mediated alterations in the binding of CcmR to DNA could still be observed, as discussed in the next section.

### SPR screening identifies α-KG and NADP^+^ as metabolic effectors of CcmR

**Figure 3:**
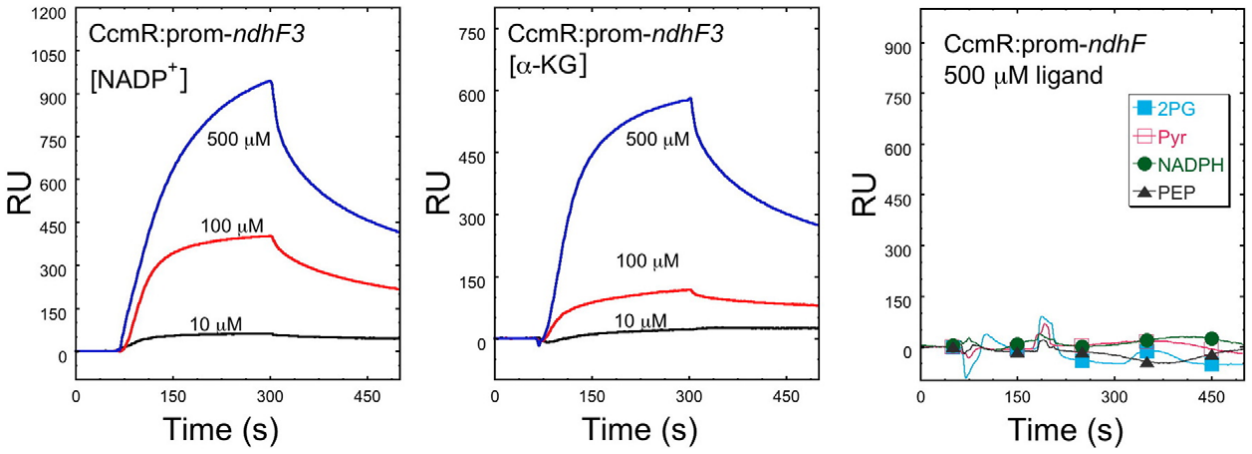
Surface Plasmon Resonance difference curves (double reference) illustrating protein binding to immobilized biotinylated DNA fragments with increasing concentrations of ligand molecule. DNA immobilization as described in Fig. 2. CcmR binding to pndhF3 fragments as the binding target. For assay method see Fig. 2. All curves are double referenced such that the curve corresponding to CcmR without added effector is subtracted from the curves corresponding to CcmR with the tested effector [33]. Proteins where incubated with the indicated ligand molecule on ice for at least 5 minutes before injection. Injections testing contain 1.5 µM of CcmR and 10 µM (Black), 100 µM (Red), or 500 µM (Blue) of the indicated ligand molecule in the case of NADP^+^ and α-KG, or 500 µM for the non-effector molecules tested. These results have allowed for the identification of NADP^+^ and α-KG as the ligand molecules for CcmR. Based on their affect on CcmR (increasing signal) these molecules function as co-repressors within the regulation system.

The activity of an LTTR is typically modulated by the binding of small molecule(s) capable of causing allosteric structural changes and changes in the DNA-binding characteristics of the LTTR [26]–[29]. Such small molecule effectors thereby act as signals allowing LTTRs to control gene expression in response to specific metabolic and environmental cues. Previous work has suggested two main hypotheses for the possible effector molecules for the regulators of the high-affinity CCM; one that they directly respond to the intracellular Ci and the other that they are directly sensing photorespiratory intermediates [1], [32]. Indeed, electrophoretic mobility shift assays were used to identify ribulose bisphosphate (RuBP) and phosphoglycolate (2-PG) as effectors of another CCM regulator, CmpR, the activator of the ABC-type bicarbonate transporter encoded by the *cmp* operon [20]. However, attempts to use electrophoretic mobility assays for identifying the effectors of CcmR proved problematic in our hands. We therefore used SPR to screen different biologically relevant molecules in carbon fixation and C_i_ transport in an effort to determine the ligand molecule for CcmR. SPR sensors were prepared with immobilized duplex DNA fragments consisting of the upstream region of the *ndhF3* operon from ­333 bp to ­191 bp relative to the translation start site (pndhF3­2) were used to screen for potential effector ligand molecules for CcmR. As noted, the sequences had been previously mapped to contain the CcmR binding regions [26] ensuring the possibility of an authentic ternary regulatory interaction between CcmR, the operator DNA, and an effector molecule. A baseline for the CcmR binding for pndhF3­2 was established by injection of 1.5 µM of the transcriptional regulator in the absence of putative ligand. The surface of the SPR biosensor was washed to remove bound transcriptional regulator and the binding of the same concentration of CcmR to pndhF3­2 was then tested in the presence of different possible effectors including HCO_3_^−^, 2-phosphoglycolate (2­PG), NADPH, NADP^+^, pyruvate, phosphoenolpyruvate (PEP), and α­KG, RuBP. To illustrate the impact of effectors on the CcmR-interaction, the curves for basal binding in the absence effector is subtracted from the curves for CcmR binding in the presence of the tested effectors producing a binding difference curve [33]. Of the molecules tested, CcmR only showed modified binding only in the presence of NADP^+^ and α-KG (Figure 3, left and middle). Maximal effects for each effector ligand were observed at 500 µM. The effective concentrations of NADP^+^ and α-KG are in the range of metabolic fluctuations in cyanobacteria [34], [35]. Increased binding of CcmR to target DNA was not observed for any of the other potential effectors tested, including NADPH (Figure 3, right). Similar results were obtained with the previously autoregulatory region of ccmR (not shown). Because NADP^+^ and α-KG enhance the binding of the repressor CcmR, we conclude that these effectors function as co-repressors. As discussed below, this conclusion is consistent with the expected behavior of these two metabolites, at least during the early phase of C_i_ limitation. Additionally, we used SPR to confirm the previous finding that binding CmpR to the upstream sequence of the *cmp* operon was stimulated by RuBP and 2-PG (Figure S3). CmpR is an activator of the *cmp* operon encoding the ABC-type bicarbonate transporter and its enhanced binding due to its interaction with RuBP and 2-PG also makes physiological sense since these metabolites are also expected to increase during C_i_ limitation.

**Figure 4:**
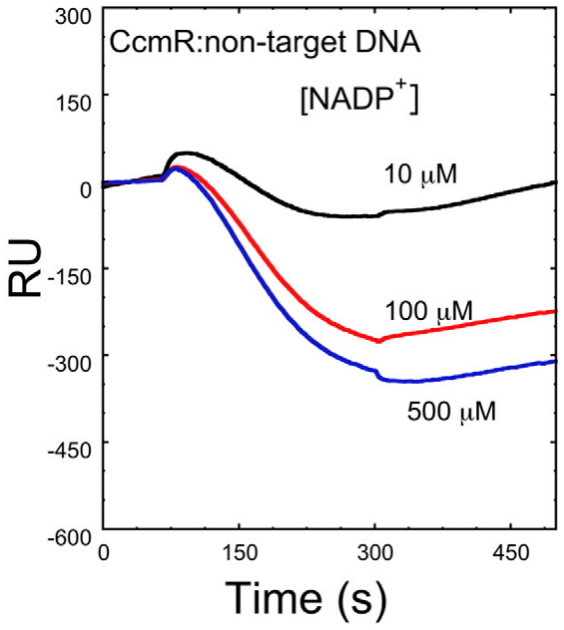
SPR difference curves showing destabilization of CcmR binding to non-specific DNA (*rimM*) due to interaction with effector molecule, NADP^+^. DNA immobilization as described in Fig. 2, except that a biotinylated 150 bp DNA fragment the *rimM* gene (non-specific DNA) was immobilized upon the SPR surface. All curves are double referenced as in Figure 3 [33]. The CcmR protein was injected at a concentration of 1.5 µM following incubation with ligand molecule on ice for at least 5 minutes before injection.

Having identified NADP^+^ and α-KG as cognate effectors of CcmR, we then tested their effect on the interaction of CcmR with non-specific duplex DNA. Using the RimM DNA fragment that had been used as a non-specific competitor in EMSAs (Data S1 Figure S1), we found that NADP^+^ and α-KG actually diminish the binding affinity of CcmR to non-target DNA, as shown in Figure 4. Thus, in contrast to causing a stronger interaction between CcmR and DNA as in the case of the target promoter sequence, a weakening of binding occurs at non-target DNA sequences when CcmR interacts with its cognate effectors. Therefore we conclude that effector binding not only enhances the binding of CcmR to its target promoters, the binding of effector produces a structural change that also increases the sequence specificity of the interaction.

In addition, we have also used SPR to confirm that 2-phosphoglycolate (2-PG) and ribulose bisphosphate (RuBP) enhance the binding of CmpR to the operator region of the *cmp* operon (Data S1, Figure S2). CmpR is homologous to CcmR and serves as an transcriptional activator for the *cmp* genes encoding the ABC-type bicarbonate transporter which had been discovered earlier by the Omata group [9], [36] and analyzed using gel shift analysis [20].

### The level of NADP^+^ present during treatment with ethoxyzolamide (EZ)

**Figure 5:**
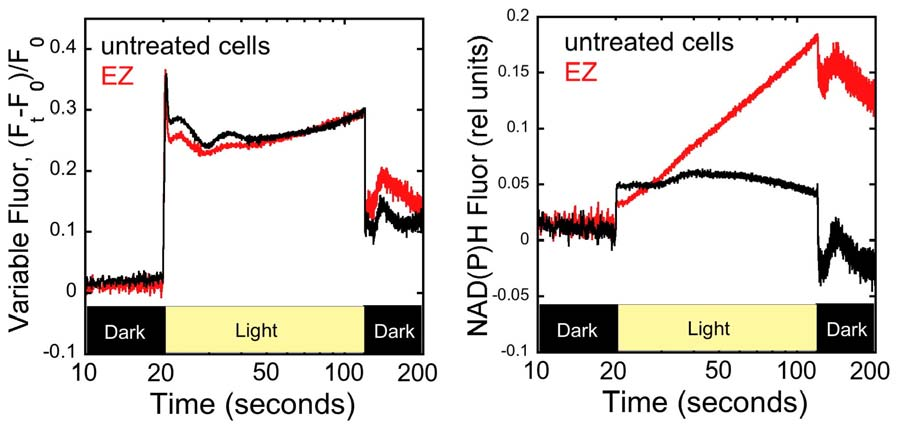
Effects of metabolic inhibitors on the redox state of the plastoquinone and pyridine nucleotide pools. Simultaneous measurements of chlorophyll *a* fluorescence (left panel) and NAD(P)H fluorescence (right panel) were made during exposure to light with an intensity approximating growth illumination (∼80 µmoles photons m^−2^ s^−1^) with *Synechocystis* cells treated C_i_-uptake inhibitor EZ (ethoxyzolamide), red traces or no addition, black traces. Note that the data are presented using a log scale for the time axis. Measurements were made using a pulse amplitude modulated (PAM) fluorometer (DUAL-PAM-100, Walz) and an emitter-detection-cuvette assembly (ED-101US) with a DUAL-ENADPH emitter (Walz) detection of chlorophyll and NADPH fluorescence (see Methods for details).

To begin to establish the connection between photosynthetic metabolism and the observed regulatory features of CcmR, we next sought to evaluate the effects of inhibitors of C_i_ uptake on one of the inferred regulatory metabolites, NADP^+^. Woodger and colleagues demonstrated that specific inhibitors of C_i_ uptake induce genes associated with the high affinity CCM [32]. These include the same genes as those repressed by CcmR [11]. Since the above SPR results indicate that CcmR repression is partly mediated by NADP^+^, it is anticipated that C_i_-limitation conditions will coincide with decreased NADP^+^ concentrations in the cell. While this supposition is also expected since limitation of carbon fixation should result in the accumulation of NADPH at the expense of NADP^+^, we nevertheless endeavored to explicitly demonstrate a relationship between decreased levels of NADP^+^ and inhibitors of C_i_ uptake. Ethoxyzolamide (EZ) is a carbonic anhydrase inhibitor that blocks the CO_2_-hydrating activity of the NDH-I_3_ system and reduces internal C_i_-pool size [37], [38] and has been shown to induce the expression of members of the CcmR regulon [37]. Specifically, EZ has been shown to disrupt the activity of the NAD(P)H dehydrogenase (NDH-I_3_) dependent CO_2_ uptake system carbonic anhydrase-like activity as part of their uptake mechanism while having little to no effect on the carboxysome carbonic anhydrase [38], [39]. We hypothesized that addition of EZ will block consumption of NADPH by the carbon fixation reactions and result in the accumulation of NADPH. Spectroscopic tools exist to probe the redox level of pyridine nucleotides in vivo [40], [41]. A modulated fluorometer, the Dual-PAM-100 (Heinz Walz GmbH), was configured for concurrent detection of chlorophyll *a* (Chl) and NAD(P)H fluorescence allowing the monitoring of both the redox state of the plastoquinone pool and the relative level of NAD(P)H within the cells of *Synechocystis*. As shown in Figure 5, Chl and NAD(P)H fluorescence traces (right and left panels, respectively) were recorded in cells that had been grown under HC conditions (3% CO_2_ supplemented air) and subjected to no chemical inhibitor treatment (black traces), treatment with 200 µM EZ. Cells were incubated in the dark for 15 minutes, the fluorescence monitors were turned on and, after 20 seconds of recording the dark samples, the cells were illuminated for 100 seconds at growth-light intensities to drive photosynthesis, this actinic illumination was switched off to allow recording of post-illumination changes in fluorescence yield for an additional 80 seconds. EZ produces little change in the yield of chlorophyll fluorescence during these brief illumination periods, which is taken to indicate that the redox state of the plastoquinone pool of the photosynthetic membranes does not become over-reduced during illumination under EZ treatment. On the other hand, pyridine nucleotide fluorescence (NAD(P)H = NADH+NADPH) increases monoexponentially during the illumination period (note the traces are plotted along the a log_10_ time axis). While the present method does not allow discrimination of NADPH versus NADH, the result is consistent with the progressive light induced reduction of NADPH at the expense of NADP^+^. Thus, the conclusion that NADP^+^ is a co-repressor of the high affinity CCM is consistent with the observed behavior of the redox response of the pyridine nucleotide system in response to C_i_ deprivation.

## Discussion

While considerable progress has been made on the structural aspects of the CO_2_-concentrating mechanism (CCM), an understanding of the regulation of the CCM has remained more elusive, especially regarding the cellular mechanisms signaling the status of C_i_ availability. The present results provide insight into the transcriptional control of the inducible C_i_ transporters by the LysR-type transcriptional regulator CcmR in *Synechocystis*. Earlier, Figge et. al. [17] used EMSA, DNA footprinting, and β-galactosidase transcriptional fusion assays to define the operator regions for CcmR binding upstream of its own gene, ccmR (sll1594) and upstream of the CUP operon gene, ndhF3 (sll1732). That work has been crucial since it provided the location of verified regulatory DNA sequences used here for the purpose of developing the SPR screening approach to identify putative co-repressors of the CcmR protein. Using SPR, it is now shown that the CcmR binds to these two operator regions and that this binding was increased by the presence of the small molecules, NADP^+^ and α-KG.

### Model for the regulation of the high affinity C_i_-concentrating mechanism

**Figure 6:**
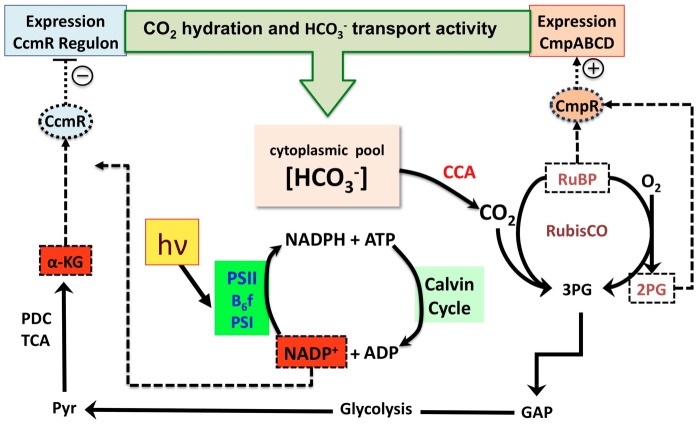
Diagram of the proposed regulatory network within Synechocystis sp. PCC6803 showing both CcmR and CmpR, along with their ligand molecules and the relevant metabolic pathways. Enzyme/complex or metabolic pathways involved in the given step are indicated in solid lines, regulatory interactions are indicated in dotted lines. [HCO_3_^−^]_cyt_,, cytosolic bicarbonate; CCA, Carboxysome Carbonic Anhydrase; PDC, Pyruvate Dehydrogenase Complex; TCA, Tricarboxylic Acid Cycle; PSII, Photosystem II; B_6_f, Cytochrome B_6_f complex; PSI, Photosystem I. Ligand molecules for transcriptional repressor CcmR (NADP^+^ and α-KG) are indicated in red boxes, while those of transcriptional activator CmpR (RuBP, 2-PG) are indicated in white boxes. For C_i_ uptake genes repressed by CcmR, see Figure 1 and reference [11]. For more information on the CmpR effectors, see reference [20] and Data S1, Figure S2.

Based upon the results presented above and those of the Omata group [20], it is possible to formulate a very preliminary model of the control of the high affinity C_i_-uptake genes of the CCM as shown in Figure 6. Before discussing the features of this model, it is important to note what the model is not taking into account and that this model will prove to be an oversimplification. At least two important findings spring to mind. First, recent work by the Kaplan group has shown that the operator region of the high affinity Na^+^-HCO_3_^−^ symport, SbtA/B, is regulated by an AbrB-type of transcriptional regulator (Sll0822) that has a repressor type of activity, which operates in addition to the repression of *sbtA/B* transcription by CcmR [11] (also see Figure 1). Already earlier work had provided a clue to a complex regulation of *sbtA/B* since RT-PCR experiments showed that transcription from the *sbtA/B* operon was completely repressed under HC conditions (3% CO_2_ enriched air), but repression of the transcription from *ndhF3* (leading gene of the CO_2_ hydration system, sll1732-sll1735 operon) was incomplete [11]. In the same experiment, both of *sbtA/B* and *ndh-I_3_* operon operon exhibited increases in transcript abundance upon a downshift in C_i_ availability and both these operons exhibited aberrant de-repression upon deletion of the *ccmR* gene. This is consistent with the existence of a two-tier repression system involving both the Sll0822 and CcmR, at least for the *sbtA/B*. Another important recent finding is there are antisense-RNA species for the initial part of the mRNA transcript for the sll1732-sll1735 operon [42], [43]. At this stage the functional significance of this antisense RNA has not been determined, but it does alert us to the possibility of additional complexity beyond the basic model proposed here. Conceivably, the complex regulation of the high affinity CCM may reflect tuning of the transcriptional responses to the relative availability of different forms of C_i_, either dissolved CO_2_ or bicarbonate. Whatever the case, the model presented in Figure 6 is considered a reasonable starting point as suggested next.

Figure 6 represents a working model of the regulation of the inducible C_i_ transporters by the LysR-like transcriptional regulators CmpR and CcmR. When C_i_ is not limiting, the constitutive C_i_ transporters BicA and NDH-I_4_ are successful at increasing the level of internal C_i_ to keep the ratio of CO_2_/O_2_ at a high level around the catalytic site of Rubisco, which is sequestered within the carboxysome. The predominant reaction catalyzed by Rubisco during these conditions is the carboxylation of RuBP to 3­phosphoglycerate (3-PG), which is converted to glyceraldehyde 3­phosphate (GAP). Correspondingly, the wasteful oxygenase reaction involving the oxidation of RuBP to form 2­PG is decreased to a very low level. [for recent results and discussion on these photorespiratory processes in *Synechocystis*, see references [22], [23], [44]]. Glyceraldehyde 3-phosphate can be built up into six carbon sugars or used to regenerate RuBP for the CBB cycle. Part of the newly fixed organic carbon is shunted to the oxidative Krebs cycle creating α-KG. Because of the lack of the α-KG dehydrogenase complex, α-KG is utilized mostly to supply a carbon skeletons for nitrogen assimilation [45], [46]. The photosynthetic reduction of NADP^+^ to NADPH is continuously taking place, yet NADPH is being rapidly utilized in carbon fixation and other metabolic processes tending to decrease NADPH/NADP^+^ ratio. Thus, when C_i_ is abundant, the high assimilatory activity of the CBB cycle keeps NADP^+^ and α-KG levels relatively high (but see below). These conditions will tend to maintain CcmR bound to its cognate repressor control DNA sequences of the C_i_ uptake genes thereby repressing their transcription. Thus, relatively high levels of NADP^+^ due to active utilization in carbon fixation and catabolism leads to active repression of transcription of the inducible transporters NDH-I_3_ and SbtA by the repressor action of CcmR in the presence of its co-repressors, α-KG and NADP^+^.

The involvement of α-KG in the control of the expression of the high affinity CCM provides a (partial) explanation for the observed coordination in global C and N assimilation gene regulation that is observed during changes in C_i_ availability [11], [47], [48]. If internal concentrations of ammonium is non-limiting, the level of α-KG would be low suggesting that NADP^+^ is a primary signal responsible for transcriptional repression of the CcmR operon in non-limiting C_i_ conditions is NADP^+^.

The other LysR-type transcriptional regulator, CmpR has also been characterized with respect to its regulation in the cyanobacterium *Synechococcus* sp. PCC7942. CmpR is an activator of the ABC-type bicarbonate transporter BCT1 and was shown to have enhanced binding to the activator sequences in the presence of 2­PG and RuBP resulting in transcription of the BCT1 transporter structural genes. We confirm that the ortholog of CmpR in *Synechocystis* performs the same way: SPR analysis using CmpR and the DNA fragments containing the upstream region from −275 bp to +25 bp of the *cmp* operon from *Synechocystis* showed specific binding of CmpR to the DNA fragment and indicated that the presence of 2-PG and RuBP increased binding (Data S1, Fig. S3). The low levels of 2­PG and RuBP due to a high level of carboxylase activity of Rubisco means that CmpR is unable to effectively activate the cmp operon leading to low accumulation of the BCT1 transporter under these C_i_ replete conditions.

Upon a shift to C_i_-limiting conditions, the constitutive transporters are unable to maintain adequate inward fluxes of C_i_, which causes a decrease in the internal concentration of C_i_. The predominant reaction of Rubisco shifts from carboxylation toward oxygenation of RuBP leading to the accumulation of 2­PG and a decrease in GAP and other organic carbon skeletons including the formation of α-KG, which is still being utilized in nitrogen assimilation. The abundance of NADPH would increase as NADP^+^ is still being reduced by photosynthetic activity while utilization of NADPH is decreased by the lack of active carbon metabolism, together leading to a relatively higher NADPH/NADP^+^ ratio. This corresponds to relatively low levels of co-repressor NADP^+^, potentiating the de-repression of the high affinity C_i_ uptake genes (Figure 6). Also, the high levels of 2­PG leads to the active transcription of the *cmp* operon through the active binding of a ligand bound CmpR, as previously suggested [20]. The utilization of RuBP in the oxygenase activity of Rubisco suggests that the level of RuBP would not increase in conditions of limiting C_i_, suggesting that 2­PG is the primary signal responsible for activation of the *cmp* operon. The higher NADPH/NADP^+^ ratio and the continuing utilization of α­KG leads to de-repression of the CcmR regulon as CcmR is no longer bound to its co-repressors. This is supported by the prerequisite of light for the expression of the inducible C_i_ transporters [5], [49], [50]. The active transcription of the inducible C_i_ transporters leads to a recovery of the internal C_i_ levels and subsequently an increase in the carboxylation activity of Rubisco. Future work should investigate the extent to which the co-repressors α-KG and NADP^+^ interact in their effects on the CcmR binding to see whether the effects are synergistic or not.

Recent metabolomics analysis of *Synechocystis* cells subjected to a shift from high to low C_i_ produced a wealth of information on the changes of metabolites following a C_i_ downshift [22]. Surprisingly from the standpoint of the present results, it was shown that α-KG actually increases 10–20 fold in concentration at the time point 3 hours following the transition to low C_i_, gradually declining after 24 hours, but still not to the pre-downshift levels. These workers also identified a reciprocal decrease in glutamine levels accompanying the increase in α-KG levels [22]. This was interpreted as being due to a block in nitrogen assimilation that occurs predominantly via glutamine synthase (GS) and the GS-GOGAT system [reviewed in reference [46]]. Lowered glutamine levels could also be attributed to other factors such as decrease growth rate and N-assimilation down-regulation due to feedback from increased ammonia levels produced by greater flux through the photorespiratory pathway, and the regulatory activity of the PII protein [22]. Regarding the blockage of N-assimilation via the GS-GOGAT system: N-status in cyanobacteria is sensed by the level of α-KG, the concentration of which is positively correlated with the expression of N-assimilation genes such as the GS [51]. This is functionally rational since α-KG is the principal carbon skeleton utilized for the assimilation of ammonia. Correspondingly, decreased levels of α-KG result in the repression of N-assimilation genes. Furthermore, decreased levels of α-KG cause the de-repression of glutamine synthase inactivating factor (GIF), which is controlled by NtcA (also a LysR-type regulator), has also been shown to bind α-KG as a co-repressor of the *gif* genes. These gene expression responses occur within minutes of the physiological transition eliciting α-KG -mediated the response. This de-repression of *gif* genes resulting from lowered α-KG concentrations thus results in a very rapid inactivation of GS that is only reversed by a proteolytic destruction of GIF that occurs once N-assimilation conditions are restored [51]. Ammonium addition results in a strong decrease in α-KG and causing the *gif* genes to be de-repressed [52]. However, those studies also showed that the shutdown of N-assimilation due to the decreased α-KG levels subsequently gave way to increased levels of α-KG because its consumption was diminished. This post-decrease restoration of α-KG levels, in turn, caused the re-repression of the *gif* genes by NtcA. Thus, the decreased α-KG levels and the concomitant de-repression of the *gif* genes was a transient event that had run its course in the tens of minutes time frame. Interestingly, the genes for GIF exhibited the same very fast (minutes time frame) up-regulation upon C_i_ downshift [11] providing circumstantial evidence that α-KG decline occurs, at least transiently, in the very early stages of C_i_ downshift and, by analogy, the α-KG may increase afterwards due to the shut-down of N-assimilation. While these inferences remain to be proved, they may account for the apparent discrepancy between the observed increase in α-KG at the 3 and 24 hour time points following C_i_ downshift [22] and the decline in α-KG levels expected based upon the regulatory behavior of CcmR and also point to the necessity to perform more detailed studies on the regulatory interactions between the α-KG and NADP^+^ in relation to their combined effects on CcmR activity and also the need to determine the possible transient changes in the early times after C_i_ downshift. Furthermore, the potential role of other metabolites, such as bicarbonate itself, has not yet been excluded. Correspondingly, a more comprehensive understanding of the CCM will require a determination of how CcmR is integrated with other regulators, including the recently discovered transcription factor Sll0822.

## Materials and Methods

### DNA Fragments and Protein

All PCR reactions were carried out utilizing recombinant *Taq* polymerase isolated essentially as previously described [53]. All primers were obtained from Integrated DNA Technologies (IDT). Modified 5′­biotinylated oligonucleotide primers were obtained from IDT for use in SPR analysis. The DNA fragments produced by the PCR reactions were concentrated by ethanol precipitation [54] and dissolved in 10 mM Pipes pH 7.4, 300 mM NaCl for use in SPR analysis. The fragments were run on a 1% agarose gel to confirm successful purification and determine if the correct length was obtained. Concentrations of the DNA fragments were determined by spectroscopic means.

Recombinant N-terminally His-tagged CcmR was purified using modified protocols from Qiagen (see Data S1, Figure S1). Protein concentration was determined spectroscopically and subsequently aliquoted (10 mM Na_2_HPO_4_ pH 8.0, 300 mM NaCl, 30% Sucrose) and snap frozen with liquid nitrogen. This is detailed in Data S1.

### Electrophoretic Mobility Shift Assay (EMSA)

EMSA reactions were run using modified protocols from the manufacture (Invitrogen) essentially as previously described [55], [56]. Briefly, the binding reactions were incubated at room temperature for 20 minutes and centrifuged at 14,000×g for 5 minutes prior to loading. Samples (30 µL) were then loaded onto 6% native­PAGE gels (50 mM Tris­OH, pH 8.5; 380 mM Glycine; 1.9 mM Na_4_-EDTA) and electrophoresed at 125 volts for 60 minutes at room temperature. The PAGE gels were post-stained with ethidium bromide and imaged (GelDoc-It, TS Imaging System).

### Surface Plasmon Resonance (SPR)

SPR was carried out using the SensiQ (ICX Technologies). Pre-coupled Neutravidin chips were obtained from ICX Technologies for use with 5′­biotinylated DNA fragments. Biotinylated DNA fragments were dissolved in immobilization buffer (10 mM Pipes pH 7.4, 300 mM NaCl) and injected on to the neutravidin surface at 5 µL min^−1^ for 50 minutes. DNA fragments were produced by PCR using primers described in supplemental data file, Data S1, Table S1. Injections were made until 300­600 RU of DNA was on the surface, 0.73 pg mm^−2^ of DNA per RU [57]. Protein samples were buffer-exchanged through the use of gel-filtration spin columns (P6DG resin, BioRad) into running buffer (10 mM Pipes pH 7.4, 300 mM NaCl, 0.02% Tween-20). Post-exchange protein concentration was determined by spectroscopic means. All centrifugations where carried out at room temperature at 2000×g for 4 minutes. The exchange columns were equilibrated using 2 washes of 100 µL each with SPR running buffer. Once the columns were equilibrated, protein samples (75–80 µL) were applied to the resin surface and centrifuged as before.

CcmR was injected into the system at 25 µL min^−1^ for 240 seconds in the presence or absence of putative ligand molecules which where incubated with CcmR for at least 5 minutes on ice before injection into the system. The interacting surface was regenerated using regeneration solution (10 mM Pipes, pH 8.5; 1 mM Na_4_­EDTA).

### Chlorophyll a fluorescence and NAD(P)H fluorescence measurements

Simultaneous measurements of chlorophyll a fluorescence and NAD(P)H fluorescence were made using a pulse amplitude modulated (PAM) fluorometer (DUAL-PAM-100, Walz) and an emitter-detection-cuvette assembly (ED-101US) with a DUAL-ENADPH emitter (Walz) housing the NADPH (365 nm) and Chlorophyll fluorescence (620 nm) measuring light and a LED Array (635 nm) for continuous actinic light. The attached detector heads included the DUAL-DNADPH with a filter sandwich (BG39, KV418, DT Cyan) (420–550 nm bandpass) with a photomultiplier for detection of NAD(P)H fluorescence and the DUAL-DR with a PIN photodiode for measuring chlorophyll (Chl) fluorescence changes.

Cells were prepared by harvesting 250 mL of high carbon (3% CO_2_ supplemented air), mid-log-phase cells grown in BG-11 (HEPES-NaOH, pH 8.0) via centrifugation at 8,000×g for 5 minutes and resuspended in fresh BG-11 to a final concentration of 100 µg of Chl mL^−1^. The cells were placed on a rotary shaker (100 rpm) under constant illumination at room temperature. Individual samples were prepared by diluting the cells to concentrations of 3 µg of Chl mL^−1^ in a cuvette and placed within the cuvette assembly with a stir bar allowing mixing. The samples were untreated or treated ethoxyzolamide (EZ) to a final concentration of 200 µM and incubated in the dark without measuring or actinic light while mixing. The measuring lights were activated 20 seconds prior to recording. The actinic light was activated 20 seconds after start of recording and deactivated after 120 seconds after start of recording, followed by an 80 second dark period with measuring lights active before recording was terminated.

## Supporting Information

Figure S1 **SDS-PAGE illustrating a typical purification of his-tagged CcmR followed by purification using Ni^2+^-affinity chromatography and ammonium sulfate fractionation of eluate.** Uninduced (UN); Induced (IN); Purified (P).
(TIF)

Figure S2 **Specific binding of CcmR binding to the promoter DNA sequences of its own gene (pccmR-1) tested using an electrophoretic mobility shift assay (EMSA).** The results confirm the original studies by Figge et al [17]. Combinations of DNA fragments corresponding to the promoter region of the ccmR gene (−110 bp to +65 bp relative to transcriptional start site), non-specific competitor DNA (coding region of an rRNA processing protein, rimM-1), and heterologously expressed CcmR were run on 6% Native PAGE gel and stained with ethidium bromide. Binding reactions were incubated 20 minutes and subjected to gel electrophoresis at 125 V for 60 minutes. Lanes 1, 3, 4, 6, contain 20 nM pccmR-1. Lanes 2, 3, 5, 6 contain 100 nM rimM-1. Lanes 4–6 200 nM CcmR. Along the bottom of the gel, bands containing un-complexed (free) *ccmR* promoter DNA or the competing non-specific DNA fragment (rimM­1), are visible. PCR-based artifacts for the competitor DNA fragment, rimM­1, which appeared as two bands, CI and CII, that did not change in position nor relative intensity upon addition of CcmR (compare lanes 2 and 5).
(JPG)

Figure S3 **SPR confirmation that the binding of the homologous LysR-type transcriptional activator, CmpR increases its binding affinity in the presence of 2-phosphoglycolate (2-PG) and ribulose bisphosphate (RuBP) shown earlier by gel shift analysis** [**20**] **.** Omata's group had had originally identified CmpR as an activator controlling the cmp operon encoding a ABC-type bicarbonate transporter [9], [16] also which we also showed to be induced during the transition to C_i_ limitation in microarray experiments [11]. CmpR was heterologously expressed in E. coli using essentially the same approach as for CcmR. SPR difference curve showing the binding of CmpR to the promoter region of cmpA affected by 2-phosphoglycolate (2-PG, left) and ribulose bisphosphate (RuBP, right panel). See Fig. 3 in main text for details. Protein was incubated with the indicated ligand molecule on ice for at least 5 minutes before injection. All injections contain 1.5 µM of CmpR and 10 µM (Black), 100 µM (Red) or 500 µM (Blue) of the indicated ligand molecule. Left Panel: 2-PG; Right Panel: RuBP.
(JPG)

Data S1 **This file contains supplemental figures and data referred to in the main article.**
(DOCX)

Table S1 **Primers Used for Immobilized Promoter DNA on SPR chips.** Oligonucleotides used for the polymerase chain reaction synthesis of DNA fragments used for surface plasmon resonance analysis of DNA-promoter interactions. The numbers refer to the number of base pairs upstream and downstream relative to the ATG translational start site.
(XLS)
