# Supplementary figures and images for: Regulation of the Cyanobacterial CO2-Concentrating Mechanism Involves Internal Sensing of NADP+ and α-Ketogutarate Levels by Transcription Factor CcmR
Source: PLoS One. 2012 Jul 20;7(7):e41286. doi: 10.1371/journal.pone.0041286 (PMC3401165; doi:10.1371/journal.pone.0041286)

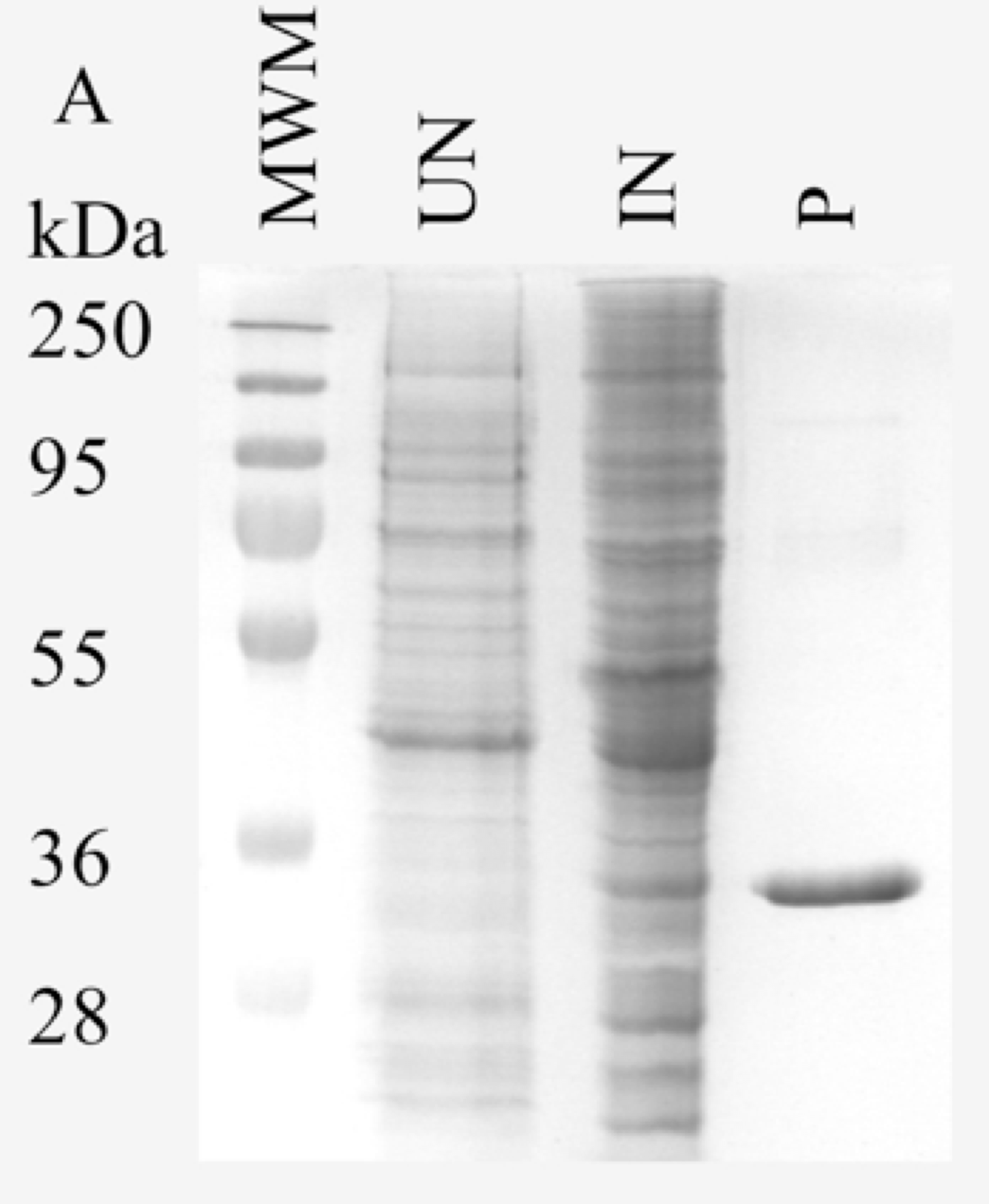

Supplement: Figure S1 — SDS-PAGE illustrating a typical purification of his-tagged CcmR followed by purification using Ni2+-affinity chromatography and ammonium sulfate fractionation of eluate. Uninduced (UN); Induced (IN); Purified (P). (TIF) [file pone.0041286.s001.tif]

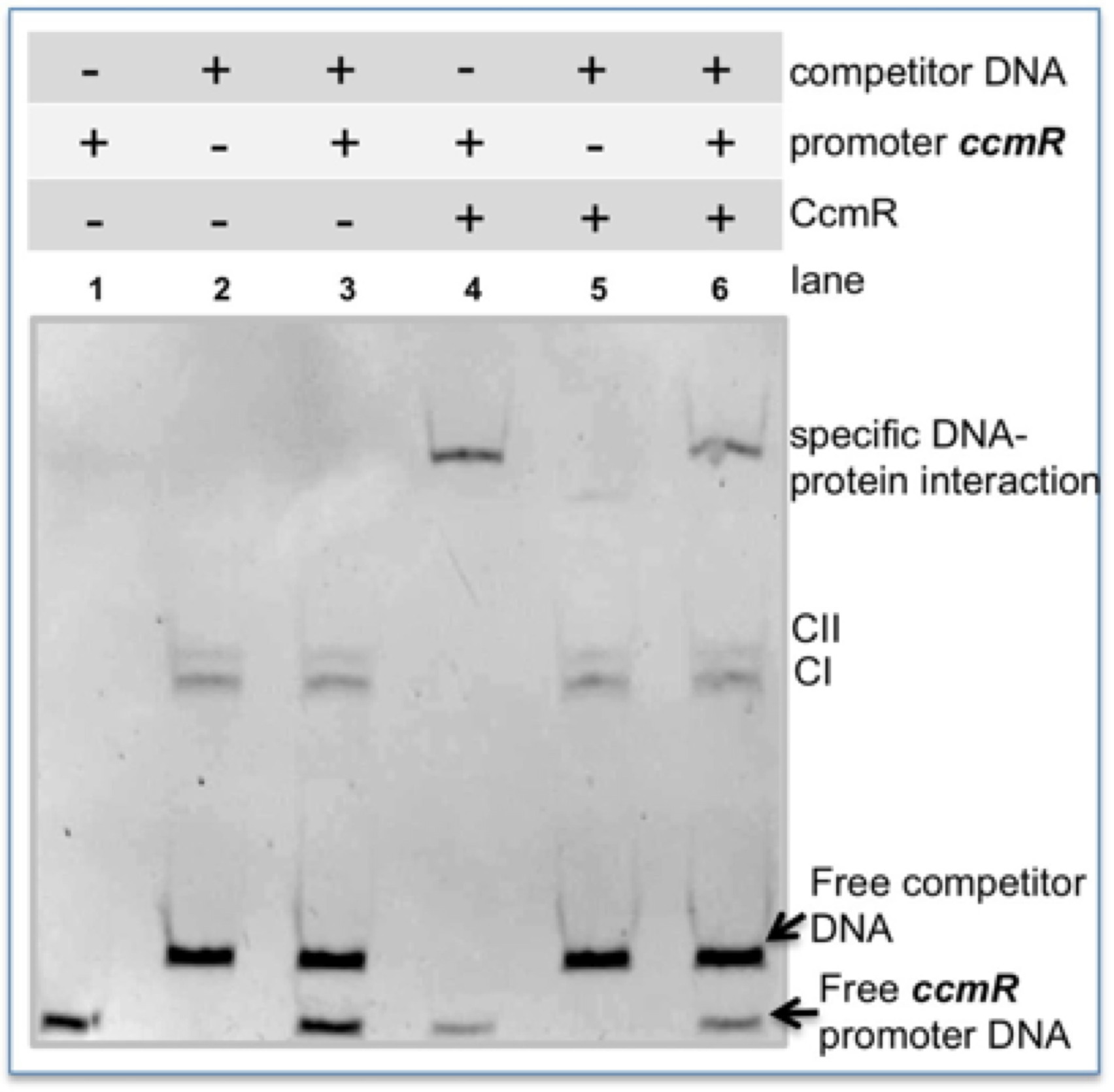

Supplement: Figure S2 — Specific binding of CcmR binding to the promoter DNA sequences of its own gene (pccmR-1) tested using an electrophoretic mobility shift assay (EMSA). The results confirm the original studies by Figge et al [17]. Combinations of DNA fragments corresponding to the promoter region of the ccmR gene (−110 bp to +65 bp relative to transcriptional start site), non-specific competitor DNA (coding region of an rRNA processing protein, rimM-1), and heterologously expressed CcmR were run on 6% Native PAGE gel and stained with ethidium bromide. Binding reactions were incubated 20 minutes and subjected to gel electrophoresis at 125 V for 60 minutes. Lanes 1, 3, 4, 6, contain 20 nM pccmR-1. Lanes 2, 3, 5, 6 contain 100 nM rimM-1. Lanes 4–6 200 nM CcmR. Along the bottom of the gel, bands containing un-complexed (free) ccmR promoter DNA or the competing non-specific DNA fragment (rimM­1), are visible. PCR-based artifacts for the competitor DNA fragment, rimM­1, which appeared as two bands, CI and CII, that did not change in position nor relative intensity upon addition of CcmR (compare lanes 2 and 5). (JPG) [file pone.0041286.s002.jpg]

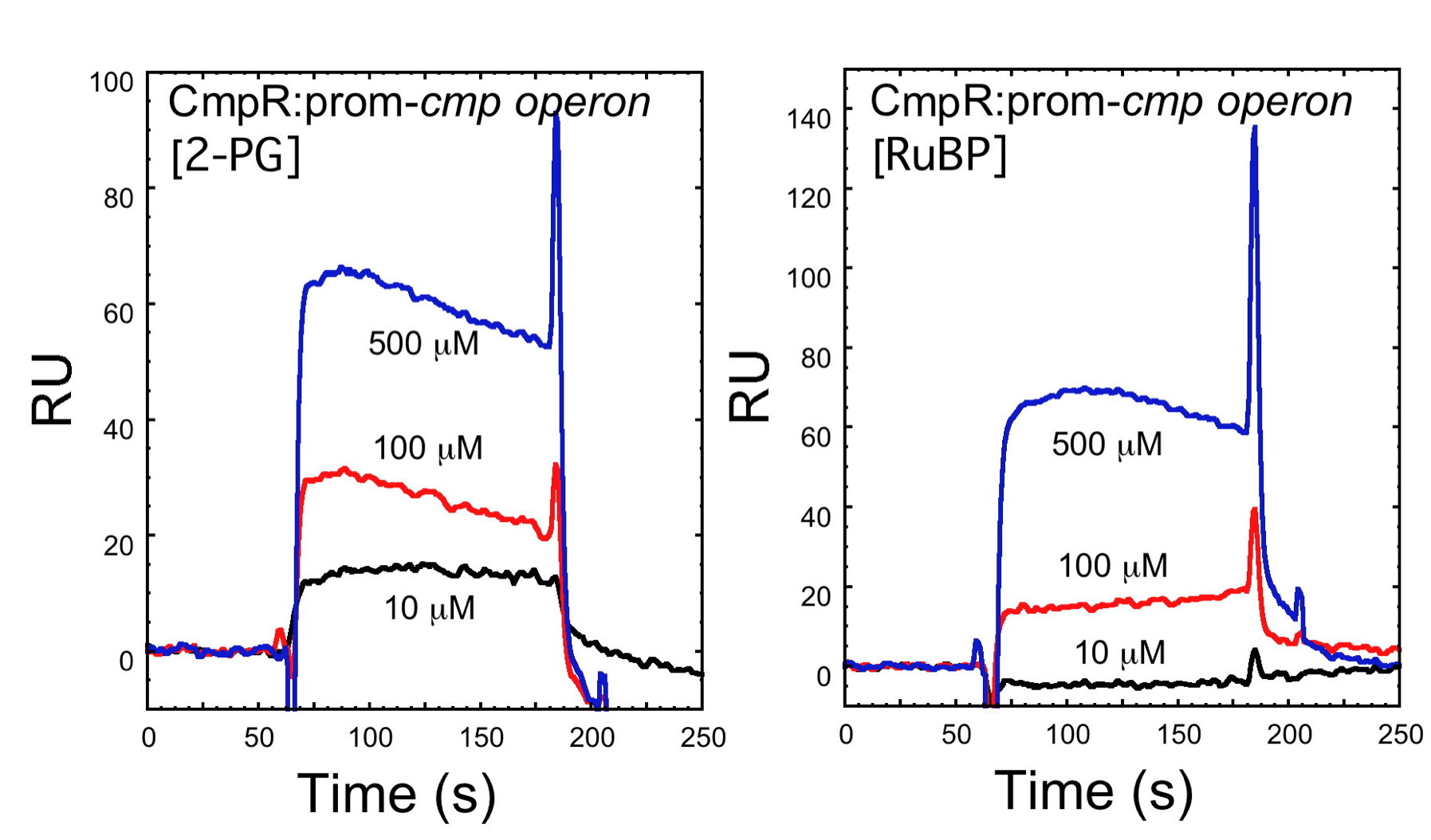

Supplement: Figure S3 — SPR confirmation that the binding of the homologous LysR-type transcriptional activator, CmpR increases its binding affinity in the presence of 2-phosphoglycolate (2-PG) and ribulose bisphosphate (RuBP) shown earlier by gel shift analysis [20] . Omata's group had had originally identified CmpR as an activator controlling the cmp operon encoding a ABC-type bicarbonate transporter [9], [16] also which we also showed to be induced during the transition to Ci limitation in microarray experiments [11]. CmpR was heterologously expressed in E. coli using essentially the same approach as for CcmR. SPR difference curve showing the binding of CmpR to the promoter region of cmpA affected by 2-phosphoglycolate (2-PG, left) and ribulose bisphosphate (RuBP, right panel). See Fig. 3 in main text for details. Protein was incubated with the indicated ligand molecule on ice for at least 5 minutes before injection. All injections contain 1.5 µM of CmpR and 10 µM (Black), 100 µM (Red) or 500 µM (Blue) of the indicated ligand molecule. Left Panel: 2-PG; Right Panel: RuBP. (JPG) [file pone.0041286.s003.jpg]
