# Supplementary material for: Regulation of the Cyanobacterial CO2-Concentrating Mechanism Involves Internal Sensing of NADP+ and α-Ketogutarate Levels by Transcription Factor CcmR
Source: PLoS One. 2012 Jul 20;7(7):e41286. doi: 10.1371/journal.pone.0041286 (PMC3401165; doi:10.1371/journal.pone.0041286)
Supplement: Data S1 — This file contains supplemental figures and data referred to in the main article. (DOCX) [file pone.0041286.s004.docx]

Supplemental Data

**Regulation of the cyanobacterial inorganic carbon concentrating mechanism involves internal sensing of NADP^+^ and α-ketogutarate levels by the LysR-type transcription factor CcmR**

Shawn M. E. Daley, Anthony D. Kappell, Marla J. Carrick, and Robert L. Burnap^*^

*Heterologous Proteins for SPR and EMSA: CcmR and CmpR expression and purification*

Protein expression of CcmR was accomplished using electrocompetent BL21 (DE3) pLysS cells (CN Biosciences, Inc.) transformed with the *ccmR* containing plasmid (see above). The transformants were plated on Luria solid media containing 100 µg/mL ampicillin, and allowed to grow overnight at 37 °C. Resulting colonies were then used to inoculate 50 mL LB in 250 mL flasks with 100 μg/mL ampicillin, and incubated overnight at 37 °C with ~250 rpm shaking. Resulting cultures were used to inoculate 1000 mL LB with 100 μg/mL ampicillin and grown with aeration at 37 °C with ~250 rpm shaking until reaching an OD_600_ of 0.4 – 0.6; when the cultures were induced with 1 mM IPTG, and allowed to incubate for a further 3 hours. Following induction cells were harvested by centrifugation, frozen with liquid nitrogen, and stored in a -20 °C freezer until needed.

CcmR was purified using the cell pellets that were stored at -20 °C. Cell pellets were lysed with the addition of 1 mL room temperature (RT) native lysis buffer (Qiagen manual) per 100 mL of cell culture volume. To ensure the compete lysis of the cells, and disruption of genomic DNA, sonication was performed with 10 – 15 second pulse durations at a power setting of 8 watts from 6 to 8 times. Following each pulse the tube was placed in an ice/water bath to be cooled for at least 1 minute before the next pulse. Following sonication the suspension was centrifuged at 14,000 x g for 5 minutes at RT. The resulting pellet was discarded and the supernatant was applied to 100 μL of Ni-NTA Superflow resin (Qiagen) per 100 mL original cell culture for affinity binding. Affinity binding was carried out in batch mode in 1.6 mL tubes (Eppendorf) by adding the supernatant from the lysis to Ni-NTA Superflow resin (Qiagen) and incubating at 4 °C for 15 – 60 minutes with gentle rocking. The suspension was spun at 14,000 x g at 25 °C for 10 – 15 seconds, and the supernatant was removed. The resin was subsequently washed 3 times with 1 mL wash buffer per 100 mL original culture volume using the same centrifugation settings. Once the washes were completed, the resin was resuspended in 1 mL elution buffer per 100 mL original cell culture with centrifugation steps as above repeated for a total of 4 elution fractions. Elution fractions were combined and ammonium sulfate was added for protein precipitation up to 30 % of final concentration. Ammonium sulfate precipitation was carried out at 4 °C with gentle rocking for at least 1 hour; then the precipitate was centrifuged at 6,000 x g for 15 – 30 minutes at 4 °C. The supernatant was discarded and the pellet was solubilized in storage buffer (50 mM Na_2_HPO_4_ pH 8.0; 300 mM NaCl; 30% Sucrose (^w^/_v_)). Protein concentration was determined by measuring absorption at 260 nm and 280 nm using spectroscopic techniques that had been calibrated for CcmR using an initial Bradford assay and the protein samples were subsequently aliquoted and snap frozen with liquid N_2_. Protein expression and purification of CmpR was carried out essentially as described for CcmR with the following modification. Following induction of the culture with IPTG the cells were allowed to incubate for a further 5 hours.

**Supplementary References**

1. Figge RM, Cassier-Chauvat C, Chauvat F, Cerff R (2001) Characterization and analysis of an NAD(P)H dehydrogenase transcriptional regulator critical for the survival of cyanobacteria facing inorganic carbon starvation and osmotic stress. Mol Microbiol 39: 455-468.

2. Nishimura T, Takahashi Y, Yamaguchi O, Suzuki H, Maeda SI, et al. (2008) Mechanism of low CO_2_-induced activation of the *cmp* bicarbonate transporter operon by a LysR family protein in the cyanobacterium *Synechococcus elongatus* strain PCC 7942. Mol Microbiol.

3. Omata T, Price GD, Badger MR, Okamura M, Gohta S, et al. (1999) Identification of an ATP-binding cassette transporter involved in bicarbonate uptake in the cyanobacterium Synechococcus sp. strain PCC 7942. Proc Natl Acad Sci U S A 96: 13571-13576.

4. Omata T, Gohta S, Takahashi Y, Harano Y, Maeda S (2001) Involvement of a CbbR homolog in low CO_2_-induced activation of the bicarbonate transporter operon in cyanobacteria. J Bacteriol 183: 1891-1898.

5. Wang HL, Postier BL, Burnap RL (2004) Alterations in global patterns of gene expression in Synechocystis sp. PCC 6803 in response to inorganic carbon limitation and the inactivation of *ndhR*, a LysR family regulator. J Biol Chem 279: 5739-5751.
